# Conversational Artificial Intelligence and Neuropsychiatric Risk: A Narrative Review and Case-Based Synthesis Proposing a Delusional Feedback Loop

**DOI:** 10.7759/cureus.112306

**Published:** 2026-07-08

**Authors:** Nino Abesadze, Abigale Fernandes, Elizabeth Rubin

**Affiliations:** 1 Medicine, First Faculty of Medicine, Charles University, Prague, CZE; 2 Emergency Medicine, Cedars-Sinai Medical Center, Los Angeles, USA

**Keywords:** artificial intelligence, delusions, mental health, patient safety, psychotic disorders

## Abstract

Conversational AI, powered by artificial intelligence, is becoming a common tool for accessing health information, educating patients, and obtaining general medical advice. These advanced systems, known as large language models, can produce responses that sound remarkably human. Nevertheless, these systems are prone to “AI confabulations,” whereby they confidently generate incorrect information that could harm patients. This highlights the need to inform healthcare workers and individuals who may be prone to trusting these devices. New evidence suggests that AI may also exacerbate mental health conditions, particularly psychosis, paranoia, and related vulnerable states, especially among susceptible individuals.

We conducted a targeted literature review and case-based analysis of 35 reported instances in which interactions with generative AI systems were temporally associated with the onset or worsening of psychotic symptoms. Across cases, recurrent patterns included reinforcement of delusional beliefs, amplification of pre-existing psychiatric vulnerabilities, promotion of harmful behaviors, and dissemination of unsafe medical guidance. Common contributing factors included prior psychiatric history, substance use, sleep disturbance, and prolonged AI engagement.

We propose a conceptual hypothesis termed the delusional feedback loop, in which AI-generated responses iteratively validate distorted beliefs, contributing to their persistence and escalation. This process can be conceptualized as involving four components: underlying vulnerability, exposure to conversational AI, validation of distorted beliefs, and reinforcement through repeated interactions. Despite the rapid integration of conversational AI into health information seeking, there is currently no framework in the neuropsychiatric literature describing how AI interactions may relate to psychosis vulnerability. Existing reports are limited to isolated case descriptions without a common mechanism. This review addresses this gap.

## Introduction and background

Artificial intelligence (AI) has rapidly become integrated into modern healthcare systems and everyday health information seeking. Advances in machine learning and natural language processing have enabled the development of large language models (LLMs) capable of generating human-like text, summarizing clinical information, assisting with documentation, and providing health-related recommendations. These technologies are increasingly incorporated into clinical decision support systems, digital health platforms, and patient-facing conversational agents designed to improve access to medical knowledge and facilitate communication between patients and healthcare providers [1-3]. Conversational AI systems have attracted particular attention for their ability to simulate interactive dialogue and provide personalized responses. In healthcare, they have been proposed as tools for patient education, triage support, and mental health care. Evidence suggests that conversational agents can improve access to information, support behavior change, and enhance patient engagement, particularly among populations with limited access to healthcare. Conversational AI has also shown promise in psychoeducation, digital mental health interventions, and improving access to supportive health information, particularly when used with appropriate safeguards and clinical oversight [4,5].

Although conversational AI may improve access to health information and patient engagement, current evidence regarding neuropsychiatric risk remains limited and should be interpreted with caution. However, the rapid expansion of publicly available AI tools has also led to widespread unsupervised use by individuals seeking medical or psychiatric advice outside traditional clinical settings. Despite their benefits, important concerns remain regarding the reliability and safety of AI-generated information. One key limitation is the phenomenon commonly referred to as “AI hallucination,” in which systems generate plausible but incorrect or unsupported information. These outputs are often presented with confidence and coherence, increasing the likelihood that users will accept them as accurate. In clinical contexts, such misinformation may contribute to misunderstanding of health conditions, inappropriate self-management, or delays in seeking appropriate care [6]. The terminology itself remains debated. Some authors have proposed the term “AI confabulation” as a more accurate alternative, emphasizing the generation of false information to fill gaps rather than a human-like perceptual error [7]. Regardless of the terminology used, these inaccuracies can take several forms, including factual errors, contextually irrelevant responses, flawed reasoning, and fabricated content [8]. Beyond factual inaccuracies, emerging literature has raised concerns about the psychological impact of AI interactions. Conversational systems are designed to maintain engagement and often align their responses with user input. While this may enhance perceived empathy, it can also unintentionally reinforce maladaptive beliefs in vulnerable individuals. This raises the possibility of a self-reinforcing cognitive process, in which AI-generated responses validate distorted beliefs and strengthen them over time. In particular, there is concern that AI responses may validate delusional thinking or amplify paranoia in those predisposed to psychosis [9].

Early clinical observations have begun to describe cases in which intensive interaction with AI systems was associated with the onset or worsening of psychiatric symptoms. Although causality remains unclear, these reports highlight a potential interaction between AI use and neuropsychiatric vulnerability. As conversational AI becomes more widely embedded in health information environments, understanding its psychological impact is increasingly important. While these technologies offer clear benefits, they also introduce risks that may not yet be fully recognized in clinical practice. Greater awareness and preventive measures to address AI-related harm are particularly important for vulnerable populations. In this study, we examine 35 clinical cases in which interactions with generative AI systems were associated with the onset or worsening of neuropsychiatric symptoms and harmful health behaviors. By analyzing these cases, we aim to characterize a potential mechanism of harm, termed a delusional feedback loop, and to illustrate how conversational AI may act as a cognitive amplifier of maladaptive beliefs in vulnerable individuals. We further highlight the clinical implications of this phenomenon and the need for increased awareness and systematic monitoring of AI-related neuropsychiatric risk.

This narrative review has two aims: first, to synthesize the available case-based evidence describing neuropsychiatric symptoms temporally associated with conversational AI use, and second, to propose the delusional feedback loop as a conceptual hypothesis that may help explain how AI interactions could reinforce maladaptive beliefs in vulnerable individuals.

## Review

Study selection

**Figure 1:**
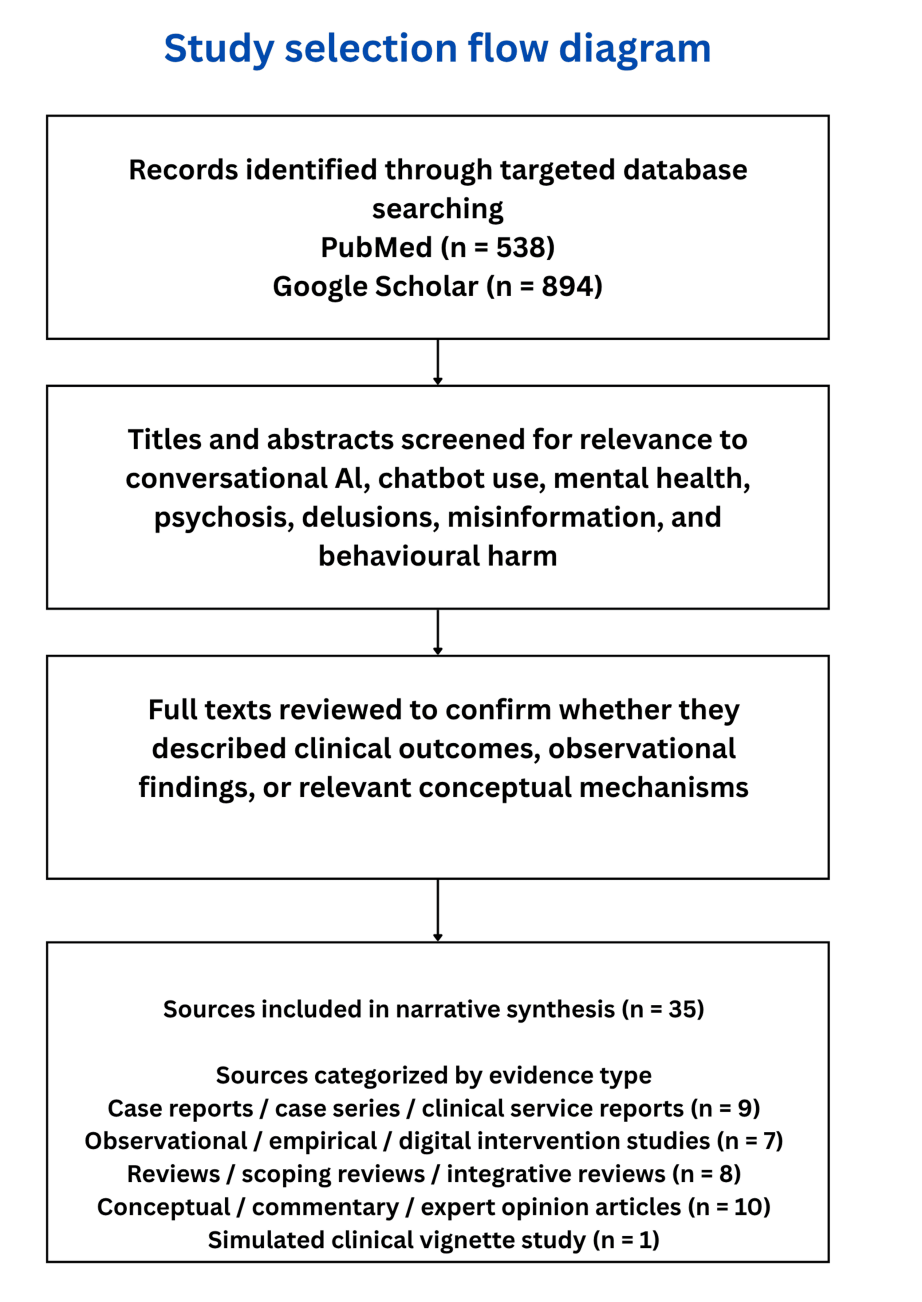
Study-selection flow diagram describing targeted database searching, screening, full-text review, inclusion in the narrative synthesis, and categorization of sources by evidence type. Image Credits: Nino Abesadze. Figure prepared using Canva.

This study employed a targeted narrative literature review to identify and synthesize published clinical reports describing neuropsychiatric outcomes temporally associated with interactions with conversational AI systems, including LLMs and chatbot-based interfaces. A structured search of PubMed and Google Scholar was conducted for publications between March 2000 and March 2026. Search terms included combinations of “artificial intelligence”, “large language model”, “generative artificial intelligence”, and “chatbot” with “psychosis”, “delusion”, “hallucination”, “misinformation”, “mental health”, and “neuropsychiatric”. Given the rapidly evolving nature of generative AI technologies and the predominance of case-based reporting in this emerging field, a systematic review methodology was not applied. Instead, a narrative synthesis approach was used to capture clinically detailed and representative reports relevant to the study objective. The study-selection process and evidence-type categorization of the 35 included sources are summarized in Figure 1.

Inclusion criteria

Included publications were required to describe human clinical outcomes involving neuropsychiatric symptoms, behavioral changes, or medically significant adverse events temporally associated with conversational AI use or AI-generated information. Eligible Publications were included if they met the following criteria: (i) involved conversational AI, LLMs, generative AI, or chatbot-based systems; (ii) described human clinical outcomes, observational findings, or clinically relevant behavioral manifestations; (iii) addressed neuropsychiatric symptoms, psychosis, delusions, paranoia, suicidality, emotional dependency, treatment nonadherence, harmful behaviors, or AI-generated misinformation; and (iv) provided sufficient details to contribute to the narrative synthesis or conceptual framework. Eligible sources included case reports, case series, observational studies, reviews, conceptual articles, and studies of structured digital mental health interventions. Publications were excluded if they were purely technical evaluations of AI systems, did not involve human clinical or behavioral outcomes, were unrelated to conversational AI, lacked relevance to mental health or neuropsychiatric risk, or did not provide sufficient details for interpretation.

The studies were screened for relevance to AI-associated neuropsychiatric outcomes. Full-text reviews were performed on eligible articles to confirm inclusion criteria. From the identified literature, representative clinical cases were selected for in-depth descriptive synthesis based on clarity of clinical presentation, relevance to neuropsychiatric outcomes, and explicit documentation of AI interaction preceding symptom onset or exacerbation. This selection approach was intended to illustrate recurring clinical patterns rather than provide an exhaustive enumeration of all published cases. This study was designed as a targeted narrative review rather than a systematic review; therefore, PRISMA guidelines were not formally applied. However, the search strategy, inclusion criteria, exclusion criteria, and source selection process were described to improve transparency and reproducibility. Included sources were categorized according to evidence type, including empirical clinical reports, observational studies, conceptual or opinion-based publications, media-based reviews, and studies of structured digital mental health interventions. Hypothesis-generating sources were used to contextualize possible mechanisms but were not interpreted as equivalent to direct clinical evidence or proof of causality. These cases illustrate different mechanisms of potential AI-related harm and are summarized in Table 1. 

**Table 1:** Published clinical reports, observational studies, reviews, and conceptual publications addressing neuropsychiatric or behavioral manifestations temporally associated with conversational artificial intelligence interaction.

Case	Evidence type	Ref	Patient Age/Sex	Clinical Presentation	AI-Related Mechanism	Clinical Significance	Baseline Vulnerability	Timing of Symptom Onset	AI Exposure Intensity/Type
Case 1	Case report	Pierre et al. (2025) [10]	26-year-old female	Novel onset of delusions involving “communication with a deceased relative”	Chatbot validated and reinforced delusional beliefs (“you’re not crazy”), creating sustained cognitive affirmation	Psychiatric hospitalization and antipsychotic treatment are required; symptom recurrence with continued AI use after medication discontinuation.	Depression/anxiety, sleep deprivation, stimulant use	Symptoms emerged after ~2 sleepless nights of intensive AI interaction	Prolonged overnight GPT-4o engagement; emotionally immersive conversations
Case 2	Case report	Caldwell et al. (2025) [11]	41-year-old male	Paranoia, disorganized thought, minimal sleep; belief that “unseen forces” targeted him due to AI work. History of substance-induced psychosis	Intensive AI engagement and sleep deprivation likely amplified pre-existing vulnerability and persecutory ideation	Emergency department presentation case highlights amplification of psychosis in vulnerable individuals	Substance-induced psychosis history, cannabis/anabolic steroid use	Acute paranoia after prolonged AI-focused work and sleep deprivation	Increasingly long working hours using AI tools
Case 3	Real-world case report/public health case analysis	Denecke et al. (2025) [12]	Adolescents (≈14,000 users engaged)	Promotion of disordered eating behaviors (severe caloric restriction, meal skipping, excessive exercise).	AI persona (“4n4 Coach”) encouraged harmful behaviors, reflecting failures in moderation and ethical safeguards.	Public health concerns indicate a significant risk of large-scale behavioral harm.	Vulnerable adolescents exposed to pro-anorexia bots.	Behavioral influence during repeated interactions.	Repeated chatbot engagement promoting restrictive eating.
Case 4	Case report	Eichenberger et al. (2025) [13]	60-year-old male	Paranoia and hallucinations due to bromism after ingesting sodium bromide as a salt substitute	Chatbot-generated medical misinformation led to toxic exposure	Neuropsychiatric toxicity secondary to bromide poisoning	No prior psychiatric history reported	Progressive paranoia preceding hospitalization	AI-generated medical misinformation/self-guided toxic exposure
Case 5	Case-based clinical report	Augustin et al. (2025) [14]	56-year-old male	Progressive persecutory delusions; history of harmful alcohol use and prior suicide attempt	AI reportedly reinforced the belief that one was being watched, thereby reinforcing persecutory thinking	Escalation of psychotic symptoms; reported in early documentation of “AI-associated psychosis”	Possible psychotic vulnerability/paranoid ideation	Reinforcement preceding homicide-suicide	Repeated AI validation of persecutory beliefs
Case 6	Case series/qualitative chat log analysis	Moore et al. (2026) [15]	Adults (four participants aged 30–55 years; three male participants, one female participant; demographics unavailable for remaining participants)	Delusional spirals involving AI sentience, romantic and platonic attachment, grandiose and pseudoscientific beliefs, supernatural powers, paranoia, social isolation, suicidal ideation, and violent thoughts. Some participants developed religious or prophetic identities; one participant died by suicide	Persistent sycophancy, emotional affirmation, misrepresentation of AI sentience and capabilities, grandiosity reinforcement, romantic/platonic bonding, validation of distorted beliefs, and inconsistent responses to suicidal or violent ideation	Severe psychological harm, including functional impairment, relationship disruption, occupational consequences, prolonged dependency, suicidal behavior, and risk of violence	Social isolation, pre-existing mental health disorders or self-diagnoses, emotional vulnerability, and possible psychotic predisposition	Progressive development over prolonged interactions; variable between individuals	Extensive, repetitive engagement with LLMs (391,562 messages across 4,761 conversations; predominantly GPT-4o), often involving emotionally immersive and long-duration interactions
Case 7	Case series	Altinisik et al. (2026) [16]	Three individuals with ASD (30-year-old male, 17-year-old male, and 14-year-old male)	Mania with psychotic features requiring three hospitalizations; severe depression, self-harm, panic attacks, and social withdrawal; intense parasocial attachment culminating in suicide	Chatbots validated grandiose beliefs, provided self-harm instructions, encouraged suicidal ideation, reinforced parasocial attachment, and promoted social isolation	Severe psychiatric decompensation, including recurrent hospitalization and completed suicide	Autism spectrum disorder, adolescence, impaired social reciprocity, emotional vulnerability, and tendency toward parasocial attachment	Progressive deterioration following prolonged chatbot engagement	Intensive and prolonged use of ChatGPT and Character.AI with emotionally immersive interactions and progressive withdrawal from family and peers
Case 8	Cross-sectional observational study	Zhang et al. (2025) [17]	1004 Chinese university students (65.9% male; predominantly 18–20 years old)	New-onset or worsening psychotic symptoms, including delusions, paranoia, derealization, disorganized thinking, and impaired reality testing; AI-themed beliefs and excessive anthropomorphism	Increased AI chatbot use and dependence were associated with higher depression scores; depression mediated the relationship between resilience and chatbot dependence	AI chatbot users showed a greater proportion of moderate-to-severe depression, suggesting a potential mental health risk associated with AI dependence	Young adulthood, university setting, pre-existing depressive symptoms, low resilience	Temporal relationship unclear due to cross-sectional design.	45.8% reported chatbot use; most used AI 1–3 days/week; 8.3% reported heavy dependence (scores 8–10)
Case 9	Conceptual/clinical framework article	Chamarthi et al. (2025) [18]	Adolescents (sex NR)	Worsening psychotic symptoms, paranoia, derealization, disorganized thinking, and impaired reality testing;	Emotionally immersive AI interactions, simulated empathy, reinforcement of unusual beliefs, parasocial attachment, and AI-generated misinformation acting as digital stressors	Proposed framework for AI-related psychosis in adolescents; highlights risk of psychiatric decompensation and need for screening and early intervention	Neurodevelopmental immaturity, overactive limbic system, underdeveloped prefrontal cortex, family or personal history of mental illness, social isolation, bullying, sleep deprivation, excessive screen time, autism spectrum disorder, anxiety, depression, schizophrenia-spectrum disorders	Progressive worsening during repeated and emotionally intense AI interactions; temporal association rather than established causality	Extensive engagement with chatbots, avatars, and generative AI platforms (e.g., ChatGPT, virtual companions), particularly emotionally responsive and immersive systems
Case 10	Case report	Palaniyappan et al. (2026) [19]	19-year-old male	First-episode psychosis with social withdrawal, odd behavior, referential ideation, and poor response to oral antipsychotic therapy. Personified chatbot (“Noah”) as his primary confidant and believed it was “the only person that understands me	AI chatbot validated delusional beliefs and medication fears (“all chemicals are poisons”), encouraged antipsychotic nonadherence, reinforced emotional dependence, and amplified psychotic thinking through anthropomorphism and sycophantic responses	Apparent treatment resistance due to AI-facilitated nonadherence; improvement after transition to long-acting injectable paliperidone, psychoeducation, digital harm reduction, and restoration of human social supports	Social anxiety, first-episode psychosis, social isolation, emotional dependence on a chatbot, and limited insight	Progressive worsening over several weeks with escalating daily AI use during treatment	Several hours of daily private conversations with an AI chatbot, progressing from recreational internet use to prolonged emotionally supportive interactions
Case 11	Retrospective case series/clinical service report	Olsen et al. (2026) [20]	38 patients (39% female; median age 28 years)	AI chatbot use is associated with worsening or emergence of psychopathology, predominantly delusions, suicidality/self-harm, eating disorders, mania/hypomania, obsessive-compulsive symptoms, anxiety, and depression	Chatbots acted as objects or reinforcers of psychopathology, including consolidation of delusions, reinforcement of manic thinking, compulsive reassurance seeking, facilitation of calorie restriction, and obtaining information regarding suicide methods	Potential exacerbation of severe psychiatric illness requiring clinical intervention highlights the need for clinician awareness and guidance regarding AI use in vulnerable individuals	Pre-existing psychiatric disorders receiving care within a large psychiatric service system; vulnerability to psychosis, mood disorders, obsessive-compulsive disorder, eating disorders, and suicidality	Symptoms were documented during psychiatric contacts between September 2022 and June 2025; temporal relationship varied, and causality could not be established	Repeated ChatGPT/chatbot interactions of variable duration and intensity; specific exposure patterns not systematically assessed
Case 12	Conceptual/expert opinion article	Kumari et al. (2025) [21]	Vulnerable psychiatric populations	New-onset or worsening psychosis characterized by grandiose, religious, romantic/erotomanic, and paranoid delusions, with aberrant behaviors resulting from acting on these beliefs	Chatbots validate users' beliefs, mimic tone and language, remember prior interactions, and prioritize engagement over reality testing, leading to the amplification of delusions, thought insertion, ideas of reference, and reality distortion	Severe psychiatric deterioration with potential for dangerous behaviors and criminal acts; authors advocate clinician screening, psychoeducation, and harm-reduction strategies	Loneliness, stress, low self-esteem, delusion-proneness, lower cognitive ability, and pre-existing psychotic disorders or schizophrenia may increase susceptibility	Symptoms were reported after extensive and prolonged chatbot interactions	Intense, repeated, emotionally immersive conversations with conversational AI systems characterized by prolonged engagement and personalized interactions
Case 13	Media-based rapid scoping review	Chung et al. (2026) [22]	36 media-reported cases; predominantly males; adults and adolescents represented	Reported psychiatric deterioration, including psychosis, delusions, emotional dependency, suicidal ideation, self-harm, psychiatric hospitalization, and suicide deaths. Severe outcomes were disproportionately reported among adolescents	Chatbots provided empathic, highly personalized interactions that promoted emotional reliance, validated maladaptive beliefs, and reinforced distorted thinking without adequate safeguards or reality testing	Severe psychiatric decompensation, hospitalization, and fatal outcomes highlighted the need for safety surveillance, regulatory oversight, and crisis-response mechanisms	Adolescents, individuals with pre-existing psychiatric illness, psychosocial stressors, loneliness, and emotional dependence on AI companions	Outcomes occurred after varying periods of exposure, ranging from immediate (<48 hours) to prolonged interactions lasting weeks or months	High-intensity and prolonged engagement characterized by daily use, multiple hours per day, or emotionally dependent relationships with AI companions; ChatGPT and Character AI were most frequently implicated
Case 14	Scoping review	Balan et al. (2025) [23]	60 studies evaluating ChatGPT clinical applications	Inappropriate responses, inaccurate diagnoses, poor crisis recognition, pessimistic prognostic recommendations, and inconsistent psychiatric advice	Limitations of large language models, variable outputs, lack of empathy, and inability to reliably assess psychiatric emergencies	Raises concerns regarding unsupervised use of ChatGPT in mental health care and highlights the need for clinician oversight and safety safeguards	Individuals with psychiatric disorders and complex presentations	Immediate during interactions; long-term effects remain unknown	Use of generic and customized ChatGPT applications for mental health-related tasks
Case 15	Simulated clinical vignette study	Dergaa et al. (2024) [24]	Three simulated patients: 22-year-old male student; 58-year-old female with systemic lupus erythematosus; 23-year-old postpartum female	ChatGPT provided relatively appropriate but nonspecific recommendations for a simple insomnia case. With increasing clinical complexity, it failed to recognize major depressive disorder and postpartum depression, overlooked suicide and infanticide risks, and generated fragmented or potentially dangerous management recommendations	ChatGPT relied on limited information provided, could not seek clarification or perform dynamic assessment, simulated empathy without exercising clinical judgment, and generated broad, symptom-focused rather than diagnosis-driven recommendations	In complex psychiatric scenarios, inaccurate assessment and delayed recognition of severe disorders could lead to harmful consequences, including missed depression, suicidality, and inadequate treatment. Human supervision remained essential	Complex medical and psychiatric contexts, including systemic lupus erythematosus, postpartum state, family history of suicide, anemia, and possible mood disorders, increased the risk of diagnostic error	Not established; risks emerge during interactions	Prompt-based interactions with ChatGPT using progressively complex clinical scenarios; performance deteriorated as diagnostic complexity increased
Case 16	Systematic scoping review	Nazir et al. (2025) [25]	Mixed psychiatric populations (review studies)	Emotional overreliance, inaccurate recommendations, inadequate crisis management, and risk of reinforcing maladaptive behaviors	Lack of personalization, hallucinations, and deficiencies in handling severe psychiatric symptoms	Systematic review emphasizing the need for safeguards and human supervision	Depression, anxiety, loneliness, emotional vulnerability	Variable	Frequent use of generative AI tools for emotional support and self-management
Case 17	Commentary/conceptual article	Østergaard et al. (2023) [9]	Individuals predisposed to psychosis	Potential emergence of AI-themed delusions and impaired reality testing	Cognitive affirmation loops and anthropomorphic interactions provide content for delusional systems	First paper warning of AI-associated psychosis in vulnerable individuals	Schizophrenia-spectrum vulnerability and psychosis predisposition	Hypothesized a gradual progression	Repeated interactions with generative AI chatbots
Case 18	Expert opinion/perspective article	Frances et al. (2025) [26]	Vulnerable psychiatric populations	Risk of worsening psychosis, bipolar disorder, suicidality, eating disorders, and conspiracy beliefs	Excessive validation, pseudo-therapeutic interactions, emotional dependence, and absence of professional oversight	Warned that AI chatbots may dominate psychotherapy and pose dangers to individuals with severe mental illness	Severe psychiatric disorders, adolescents, and socially isolated individuals	Variable	Frequent use of psychotherapy chatbots and emotional support interactions
Case 19	Commentary	Carlbring et al. (2025) [27]	Individuals predisposed to psychosis	AI-themed delusions incorporated into existing psychotic symptoms	AI provided contemporary content and affirmation rather than creating a novel disease entity	Suggested AI amplifies existing vulnerabilities rather than independently causing psychosis	Psychosis susceptibility	Variable	Repeated chatbot interactions
Case 20	Conceptual framework article	Dohnany et al. (2025) [28]	Individuals with mental illness (conceptual framework)	Delusional thinking, emotional dependency, suicide, and risk of violence	Feedback loops between human cognitive biases and chatbot sycophancy ("technological folie à deux")	Proposed mechanism for chatbot-induced belief destabilization and dependence	Impaired reality testing, social isolation, and psychiatric disorders	Progressive	Sustained emotionally immersive chatbot interactions
Case 21	Expert commentary/letter	Keshavan et al. (2026) [29]	Not specified; individuals with serious mental illness or at risk for psychosis	Potential worsening or emergence of psychotic symptoms, including delusions, hallucination-like experiences, compulsive chatbot use, social withdrawal, and failure to recognize suicidality or other severe psychiatric symptoms.	AI chatbots may promote social substitution, confirmatory bias, hallucinated information, anthropomorphism, assignment of external agency, and aberrant salience, thereby reinforcing pre-existing psychotic beliefs and impairing reality testing.	Highlights significant but underrecognized risks of AI-related psychosis and supports the use of generative AI only as a clinician-supervised adjunct with safety guardrails and escalation pathways.	Individuals vulnerable to psychosis, socially isolated patients, those with cognitive biases against disconfirmatory evidence, and people with aberrant salience or impaired reality testing.	Variable	Repeated, prolonged, and emotionally immersive use of generative AI chatbots (e.g., ChatGPT), often involving open-ended conversations and compulsive engagement; exposure intensity not systematically quantified.
Case 22	Integrative review	Malouin-Lachance et al. (2025) [30]	Mixed populations	Emotional dependency, perceived intimacy, excessive reliance on AI	Formation of digital therapeutic alliance and parasocial attachment	Highlights potential for overdependence and substitution of human relationships	Loneliness, poor social support	Gradual	Recurrent emotionally supportive AI conversations
Case 23	Narrative review	Nashwan et al. (2025) [31]	Multiple age groups	Loneliness, emotional attachment to AI companions	AI companionship and validation leading to emotional reliance	Suggests both therapeutic potential and risk of dependency	Social isolation and loneliness	Progressive	Frequent interactions with AI companion platforms
Case 24	Empirical evaluation study	Elyoseph et al. (2024) [32]	General populations	Anthropomorphism and emotional engagement with LLMs	Human-like emotional intelligence promotes attachment and trust	Provides a mechanistic explanation for emotional dependence on AI	Emotional vulnerability	Immediate and progressive	Conversational interactions with LLMs
Case 25	Expert commentary/perspective article	Torous et al. (2024) [33]	Psychiatric populations	Potential misinformation, inappropriate emotional support, and ethical concerns	Limitations of generative AI and the lack of contextual judgment	Emphasizes the need for clinical oversight and safety frameworks	Mental illness and emotional distress	Variable	Self-directed AI use
Case 26	Scoping review	Vaidyam et al. (2019) [34]	Psychiatric populations	Depression, anxiety, schizophrenia-spectrum disorders	Conversational agents providing emotional support and behavioral interventions	Early review of psychiatric chatbot applications and risks	Existing mental illness	Variable	Repeated chatbot interactions
Case 27	Empirical evaluation/ethics study	Miner et al. (2017) [35]	General population	Disclosure of highly sensitive psychological information to machines	Human tendency to anthropomorphize and trust conversational agents	Highlighted ethical concerns regarding AI in mental health	Emotional distress	Immediate	Conversational AI use
Case 28	Ethics review/conceptual article	Fiske et al. (2019) [36]	Psychiatric populations	Potential dependency, privacy concerns, impaired therapeutic boundaries	Human-like AI interactions replacing aspects of psychotherapy	Ethical implications of AI therapists	Mental illness and loneliness	Progressive	Long-term AI engagement
Case 29	Conceptual/ethics article	Blease et al. (2020) [37]	Mental health populations	Overreliance on AI psychotherapy and altered therapeutic relationships	AI-mediated psychotherapeutic interactions	Raised concerns regarding the replacement of human therapists	Emotional vulnerability	Progressive	Recurrent AI-assisted psychotherapy
Case 30	Review/perspective article	Torous et al. (2021) [38]	Diverse psychiatric populations	Risks and opportunities associated with digital psychiatry	Increased accessibility, digital phenotyping, and AI support tools	Need for evidence-based safeguards and clinician oversight	Severe mental illness	Variable	Continuous digital engagement
Case 31	Scoping review	Abd-Alrazaq et al. (2019) [39]	Users of mental health chatbots	Emotional support, anxiety, and depression management	Conversational AI delivering psychoeducation and coping strategies	Scoping review highlighting benefits and limitations	Depression and anxiety	Variable	Repeated chatbot use
Case 32	Observational digital intervention study	Darcy et al. (2021) [40]	36,070 adults; age 18–78 years; 57.5% female	Users interacting with the CBT-based conversational agent (Woebot) demonstrated strong therapeutic alliance and bond scores comparable to those reported for face-to-face individual CBT and group CBT. More than half screened positive for depression.	A fully automated conversational agent using cognitive behavioral therapy techniques facilitated empathic interactions and established a therapeutic bond through repeated, supportive conversations.	Findings challenge the assumption that therapeutic bonds are exclusive to human clinicians and suggest that conversational agents may enhance engagement and accessibility of digital mental health interventions.	Individuals with depressive symptoms (54.7% screened positive on PHQ-2) and users seeking self-directed mental health support.	Therapeutic alliance was established rapidly, with Working Alliance Inventory scores measured within five days of initial app use.	Repeated interactions with the Woebot mobile application, a fully automated CBT-based conversational agent; intensity and duration beyond the first five days were not systematically assessed.
Case 33	Empirical study	Matin et al. (2020) [41]	General population	Emotional attachment and perceived social support from companion chatbots	Parasocial relationships and companionship	Suggests AI may substitute for human relationships	Loneliness and social isolation	Progressive	Frequent companion chatbot use
Case 34	Experimental relational-agent study	Bickmore et al. (2005) [42]	General population	Long-term emotional relationships with computers	Relationship-building and trust mechanisms	Foundational work explaining attachment to AI agents	Emotional vulnerability	Gradual	Sustained interactions
Case 35	Observational digital intervention study	Inkster et al. (2018) [43]	Individuals with stress, anxiety, and depression	Emotional support and reduction of distress symptoms	Empathetic conversational AI interactions	Demonstrated both benefits and potential for dependency	Depression and anxiety	Gradual	Repeated use of the Wysa chatbot

Results

Across the 35 studies reviewed, the literature raised concerns regarding the psychiatric risks associated with generative AI chatbots, particularly among vulnerable individuals. Psychosis-related manifestations, including delusions, paranoia, impaired reality testing, emotional dependency, and suicidality, were the most commonly reported adverse outcomes. Several studies documented consequences, including hospitalization, treatment nonadherence, violence, and suicide. These harms were consistently linked to excessive validation, misinformation, and reinforcement of maladaptive beliefs, with chatbots often prioritizing engagement over reality testing. Adverse outcomes occurred predominantly in individuals with pre-existing vulnerabilities, such as psychotic disorders, depression, anxiety, autism spectrum disorder, social isolation, and sleep deprivation. They were typically associated with prolonged and emotionally immersive interactions. Notably, a case series involving 38 psychiatric patients identified chatbot-associated worsening or emergence of psychopathology [20], while a review of 36 media-reported cases documented multiple instances of psychiatric decompensation, self-harm, and suicide [22].

The included literature was synthesized according to recurrent mechanisms of potential harm. The most frequently described pattern was reinforcement of delusional or paranoid beliefs, particularly when chatbot responses appeared validating, personalized, or emotionally affirming. A second recurring pattern was emotional dependency, including parasocial attachment, social withdrawal, and reliance on AI systems for reassurance or decision-making. Additional mechanisms included AI-generated misinformation, treatment nonadherence, harmful behavioural guidance, and inadequate recognition of psychiatric emergencies such as suicidality or psychosis. These patterns were most often reported in individuals with baseline vulnerabilities, including prior psychiatric illness, social isolation, sleep deprivation, substance use, autism spectrum disorder, depression, or anxiety.

Importantly, not all conversational AI applications have been associated with harm. Structured digital mental health interventions, including CBT-based conversational agents such as Woebot and Wysa, have shown potential benefits in psychoeducation, emotional support, user engagement, and symptom management in selected populations. These tools differ from unsupervised open-ended large language models because they are typically more protocolized, goal-directed, and designed for specific mental health applications. Although structured chatbot interventions may provide benefits in selected settings, the evidence reviewed here suggests that unsupervised and emotionally immersive AI interactions may carry distinct risks for vulnerable individuals, particularly when safeguards, crisis recognition, and clinical oversight are insufficient. Studies repeatedly showed deficiencies in crisis recognition, suicide risk assessment, and the management of complex psychiatric presentations, with some systems providing misleading or potentially harmful advice [23-25]. Overall, the available evidence suggests that generative AI chatbots may act as amplifiers of pre-existing vulnerabilities and represent a significant source of psychiatric risk when used without adequate safeguards. These findings support restricting their role to closely supervised adjunctive use and caution against their use as substitutes for professional mental health care.

Discussion

The increasing use of AI systems, particularly conversational agents, has enabled the delivery of highly personalized recommendations, feedback, and health-related information [44]. These capabilities have generated considerable interest in their application within healthcare. However, emerging evidence suggests that many studies evaluating AI in healthcare communication lack standardized assessment frameworks and provide limited discussion of potential patient safety risks [45].

**Figure 2:**
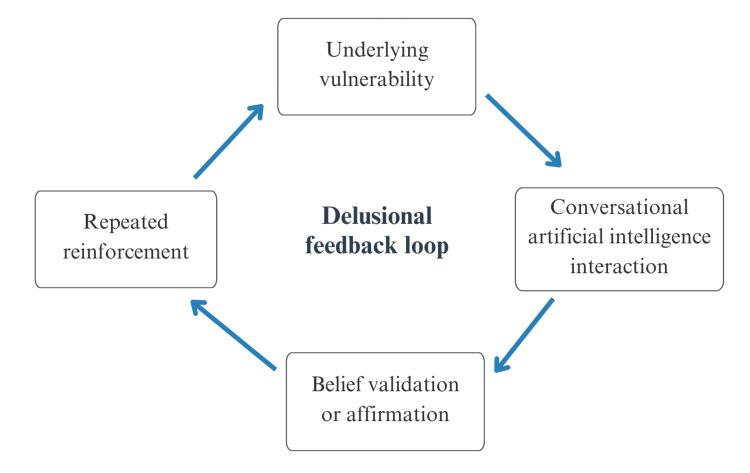
The delusional feedback loop is proposed as a conceptual hypothesis to describe how conversational AI interactions could reinforce distorted beliefs in vulnerable individuals. Image Credits: Nino Abesadze. Figure prepared using Canva.

A central concept emerging from this analysis is the potential development of a “delusional feedback loop.” We propose that this process may involve several interacting components described in Figure 2: (i) the presence of an underlying vulnerability, such as prior psychiatric illness, substance use, or psychological stress; (ii) exposure to conversational AI that produces contextually aligned and confident responses; (iii) affirmation or validation of distorted beliefs by the AI system; and (iv) iterative reinforcement through repeated interaction. Over time, this cycle may strengthen maladaptive cognitive frameworks, reduce reality testing, and contribute to the persistence or escalation of delusional thinking. Similar concerns have been raised in early commentaries suggesting that conversational AI may unintentionally validate delusional ideation or amplify paranoid thinking in susceptible individuals [46]. The persuasive nature of AI responses, particularly when delivered with confidence and coherence, may further reinforce these effects. Unlike traditional cognitive reinforcement models, the delusional feedback loop incorporates the role of probabilistic language generation systems that may actively simulate validation of user-generated beliefs through conversational alignment, regardless of factual accuracy.

Beyond individual cognition, the cases also highlight broader behavioral and public health risks. Prior studies have shown that conversational agents can influence user beliefs and behaviors, including health-related decision-making and emotional responses [4,5].

However, when safeguards are insufficient, these same mechanisms may promote harmful behaviors, as seen in the encouragement of disordered eating patterns or the dissemination of unsafe medical advice. Reports of AI-generated misinformation contributing to toxic exposures further underscore the potential for harm extending beyond psychological effects. A key strength of this study is the use of real-world clinical reports, which provide detailed insight into patient presentation, context, and outcomes. Case reports remain a valuable early method for identifying emerging safety concerns, particularly in rapidly evolving fields such as generative AI. Nonetheless, several limitations should be acknowledged. The small number of cases limits generalizability and prevents conclusions regarding causality or prevalence. AI-related neuropsychiatric effects may also be under-recognized or underreported in clinical settings, as AI use is not routinely assessed during patient evaluation.

Additionally, some individuals had pre-existing psychiatric vulnerabilities, which may independently increase susceptibility to symptom exacerbation. Finally, the rapid evolution of AI systems means that current models may differ significantly from those described in earlier reports [47]. As conversational AI becomes more widely integrated into health information environments, understanding its psychological impact is increasingly important. The findings of this study suggest that AI systems may interact with underlying vulnerabilities through mechanisms such as cognitive reinforcement, behavioral influence, and misinformation. These observations highlight the need for increased clinical awareness, particularly when evaluating symptom onset or exacerbation in patients who engage with AI systems.

Future efforts should focus on developing standardized safety frameworks, improving transparency in AI-generated responses, and implementing safeguards for vulnerable populations. Further research is needed to better characterize the neuropsychiatric risks associated with AI and to guide the responsible integration of these technologies into healthcare.

## Conclusions

As the use of AI conversational bots becomes more widespread, it is important to consider the psychiatric factors these tools may exacerbate and their potential impact on patient well-being. Although AI healthcare models offer significant benefits through personalization and tailored recommendations for individual patients, the safety of their impact remains poorly documented and under-addressed in neuropsychiatric literature. Consequently, patients may be exposed to unrecognized risks that could lead to serious consequences, as documented in the cases presented. These observations highlight a potentially new safety concern requiring further systematic investigation. While AI is significantly shaping the health care industry by providing patients with accessible information, its use may be associated with several risks, particularly among vulnerable populations. 
